# Job Loss or Income Loss: How the Detrimental Effect of Unemployment on Men's Life Satisfaction Differs by Immigration Status

**DOI:** 10.3389/fsoc.2020.00010

**Published:** 2020-02-28

**Authors:** Jing Shen, Irena Kogan

**Affiliations:** MZES, University of Mannheim, Mannheim, Germany

**Keywords:** unemployment, job loss, objective income loss, perceived financial strain, immigrant, native-born, men, life satisfaction

## Abstract

Driven by the ongoing debate of job loss vs. income loss in understanding the detrimental effect of unemployment, this study examines how perceptions of unemployment and the resulting levels of life satisfaction differ by immigration status. Based on a countrywide longitudinal dataset in the UK, findings show that immigrant men's life satisfaction suffers more from the detrimental effect of job loss *per se*, whereas that of native-born men suffers more in the pecuniary respect, which is mainly driven by perceived financial strain, instead of objective income loss. By further examining the heterogeneity among immigrant men themselves, we find similar differences between recent non-EU immigrant men and the rest of the group. While job loss causes a deeper decline in life satisfaction for recent non-EU immigrant men, income loss causes a deeper decline in life satisfaction for recent EU and established immigrant men. We attribute those differences to the extent to which one's legal status in the country is vulnerable to unemployment.

## Introduction

Based on robust evidence drawn from the German Socio-Economic Panel Study (1990–2014), in their recent publication in *Demography* Leopold et al. (2017) have argued that unemployment hurts life satisfaction of immigrant men more than that of their native-born counterparts. However, explanations about why this is the case remain unclear. This is particularly because the immigrant-native gap in life satisfaction cannot be explained by commonly used mediators of the relationship between unemployment and subjective well-being (SWB hereafter), such as the differences in socio-demographic and socioeconomic characteristics, as well as cultural values between immigrants and the native-born (Leopold et al., 2017, p. 239). The authors thus speculate that immigrant and native-born men may perceive costs of unemployment differently. To date, however, no study has directly touched upon in which exact respects perceptions of unemployment differ by immigration status.

Existing discussion about the detrimental effect of unemployment on SWB has mainly focused on two aspects: the detachment from a workplace due to job loss and the deprivation of economic resources due to the accompanying income loss (Björklund, 1985; Clark and Oswald, 1994; Korpi, 1997; Winkelmann and Winkelmann, 1998; Creed and Reynolds, 2001; Ervasti and Venetoklis, 2010; see also a review by McKee-Ryan et al., 2005). To date, much of the debate is still centered on the question “Which aspect is more hurtful to one's SWB, between job loss and income loss?” Answers to this question remain controversial, because the level of one's SWB involves complicated comparison mechanisms, so that perceptions of unemployment and the resulting levels of SWB—e.g., indicated by life satisfaction—vary from individual to individual (Campbell et al., 1976; Michalos, 1985).

Previous studies have shown that the detrimental impact of unemployment on SWB varies with individual characteristics. Scholars have generally agreed that the psychological costs of unemployment are higher for men (Lucas et al., 2004), the highly educated (Clark and Oswald, 1994), those with poorer health (Wilson and Walker, 1993), those with religious beliefs (Shen and Kogan, 2019), as well as among the middle aged compared to the young and old (Clark et al., 1996; Winkelmann and Winkelmann, 1998; Shields and Wailoo, 2002). How one perceives and feels about being unemployed is also contingent on environmental factors. For example, unemployment would be a more stressful event among those with unemployed partners compared to those with working partners (Clark, 2003), those with more, as compared to with less, dependent family members (McClelland, 2000), and those who are not or poorly protected by unemployment benefits (Clark and Oswald, 1994). To the best of our knowledge, however, there has not yet been a study focused on how the effect of unemployment on individual SWB varies in the dimension of immigration status.

We must emphasize that we will take an exclusive focus on men in the labor force, since men's labor market participation is a relatively universal phenomenon across societies. By contrast, there is a much larger degree of heterogeneity in labor force participation and its contributions to women's SWB (Leana and Feldman, 1991; Clark et al., 1996; Clark, 2003; Fahey and Smyth, 2004). Moreover, employment shifts do not seem to have a differentiated impact on life satisfaction of immigrant and native-born women (Leopold et al., 2017). All the existing findings have made it clear that the impact of unemployment on women's SWB would require a separate investigation.

Immigration status matters for men's perceptions of unemployment, because the extent to which a man's legal residence is vulnerable to unemployment directly affects in which respect(s) and to what extent he considers unemployment detrimental. Based on the social comparison theory (Campbell et al., 1976; Michalos, 1985), these subjective evaluations do not simply mirror one's factual status, but instead, are formed in comparison with relevant others. In this study, we therefore ask the following research question: Between job loss and income loss, which aspect of unemployment hurts life satisfaction of immigrant men more, in comparison with their native-born counterparts and among themselves, respectively?

## Job Loss vs. Income Loss: Detrimental Consequences of Unemployment

Scholars have long agreed that unemployment deprives an individual of multiple needs that can only be obtained through work. The term “deprivation” has become the most well-known in Jahoda's (1982) paper, which refers to distress resulting from the deprivation of five latent functions of work during unemployment; namely, time structure, social contact, collective purpose, status, and activity. As only employment can sufficiently provide these latent functions in modern societies, unemployment would unavoidably deprive the person of self-identity in a broader social setting beyond the household, subsequently causing a decrease in SWB [see also the review by Paul and Moser (2009)]. Similarly, Sirgy et al. (2001) have identified seven major needs related to work: health and safety needs, family needs, social needs, esteem needs, actualization needs, knowledge needs, and aesthetic needs. Job loss restricts one's possibilities to fulfill these needs, causing a decline in SWB. In addition, Fryer's (1986, 1995) agency theory, in which individuals are considered social actors trying to reach desirable goals, and Ezzy's (1993) theory of status package, which posits employment as a channel for one to give meaning to objective social relationships, are also influential in this line of research.

Empirical evidence from this approach has generally supported that the detrimental effect of unemployment is mainly due to job loss *per se*, and that income loss is only of secondary importance. In their studies based on the German Socio-Economic Panel (GSOEP), Winkelmann and Winkelmann (1995, 1998) decomposed the total well-being costs of unemployment into these two parts in fixed effect models. Their findings show that well-above 75 percent of the detrimental effect of unemployment was non-pecuniary resulting from job loss itself, while below 25 percent was due to income loss (Winkelmann and Winkelmann, 1995, p. 293). Also drawing data from the GSOEP, Knabe and Rätzel (2011) altered income measures by distinguishing permanent income from current income. Although the non-pecuniary costs of unemployment are reduced this way, results by and large support the importance of work in increasing life satisfaction, as the decline in life satisfaction resulting from job loss itself is still significantly larger than that due to income loss for both unemployed men and women. In addition, high costs of job loss, at the given income level, have generally been found in the United States (Helliwell and Huang, 2011; Young, 2012), the United Kingdom (Blanchflower and Oswald, 2004) and among EU citizens (Pittau et al., 2010).

In contrast with job loss, the other aspect of unemployment, income loss has remained controversial in existing literature. The loss of a stable income source cuts off one's access to sufficient food, shelter, heat, and ability to pay bills, and such worsening socioeconomic conditions would reasonably impact one's SWB negatively (McKee-Ryan et al., 2005). However, by merely highlighting the material costs of unemployment, early investigations seem to show a tendency to equate unemployment with income loss, so that there are policy suggestions aiming to reduce unemployment rates by cutting down unemployment benefits [see the review by Clark and Oswald (1994)]. Those policies are driven by the assumption of monetary returns being the only incentive for people to work. Derived from this assumption, one may intuitively think that individuals do not necessarily perceive unemployment negatively, but instead, even stay unemployed voluntarily, as long as their financial needs are satisfied. Based on the British Household Panel Study, Clark and Oswald (1994) have tested this opinion, and found that despite the financial compensation, the unemployed still have much lower levels of SWB than their employed counterparts, which suggests that the detrimental effect of unemployment cannot solely be explained by objective income loss.

The recent development of the literature has deepened scholarly understanding about income loss due to unemployment, by shifting the focus from one's objective income loss to subjective perception of income loss. In their study based on the European Social Survey from 21 countries, Ervasti and Venetoklis (2010) criticize that the detrimental effect of the financial aspect of unemployment has largely been underestimated, as previous studies took only the objective measure of income loss into account. Perceived financial strain or hardship, which indicates the extent to which one is worried about his or her financial situation and feels difficult to make ends meet, plays an important role in SWB (Ullah, 1990; Vinokur and van Ryn, 1993; McKee-Ryan et al., 2005). This relationship is independent from objective financial resources, as perceived financial strain has been found to be only moderately correlated with objective financial resources (Ervasti and Venetoklis, 2010). When objective income loss and subjective perception of financial well-being are both included in the analysis, perceived financial strain is found to explain the negative effect of unemployment on SWB much more effectively than the objective measure (Ullah, 1990). For example, financial strain is found to be the key stressor during unemployment, and one's perceptions of the current as well as future financial well-being account for 50–90 percent of psychological impact of unemployment, measured by the GHQ (General Health Questionnaire) Likert scale or other mental health problems (Kessler et al., 1988; Price et al., 2002). In Ervasti and Venetoklis's (2010) study, the inclusion of perceived financial strain reduces the negative effect of unemployment to a level of non-significance in some European countries.

In short, existing literature about unemployment has mainly focused on the debate between job loss and income loss, with the latter being further distinguished between objective income loss and perceived financial strain. The purpose of this study is thus to clarify the relative importance between the two aspects of psychological costs of unemployment on men's life satisfaction, and more importantly, how the relative importance differs between immigrant and native-born men, as well as among immigrant men themselves.

## Comparison Between Immigrant and Native-Born Men

One direct consequence of unemployment lies in the loss of economic resources to sustain a man himself and his dependents. Such a detrimental effect resulting from the loss of the major income source applies to every unemployed man, regardless of one's immigration status. However, we expect that the extent to which an adverse income change has a negative impact on life satisfaction differs between immigrant and native-born men. Immigrants are usually fully aware of difficulties of job obtainment in the host-country labor market. For example, they are often unfamiliar with labor market institutions, lack formal credentials that are recognizable in the host country, struggle to build informal ties that may lead to better jobs, and are often geographically constrained into a certain area with limited job opportunities (Elliott, 2001; Aguilera and Massey, 2003; Kogan, 2004, 2011). Thus, when unemployed, immigrants are more likely to attribute their failure in the labor market to disadvantageous circumstances associated with their immigrant status. By contrast, native-born men do not encounter many of the obstacles facing immigrants, as they are at a relatively privileged status in the socioeconomic hierarchy. This means that, when unemployed, they have fewer external reasons to draw upon to justify their income drop. As the attribution theory (Cohn, 1978) posits, the more a man is able to attribute his adverse status change to external reasons, the less painful he would perceive this change to be. On the contrary, the lack of channels of externalization naturally means an increasing tendency of internalizing the cause of the status change, which subsequently increases mental stress resulting from the change. Thus, we hypothesize that, other covariates being equal:

*Hypothesis 1: The adverse income change due to unemployment has a greater detrimental effect on life satisfaction of native-born men than that of immigrant men*.

Moving beyond objective income loss, we take a further look at how immigrant and native-born men evaluate their own financial well-being under unemployment. The sense of financial well-being is only moderately related to the objective income status as aforementioned, and it is always in the relative sense based on comparisons with one's own past experience and social comparisons with a desirable reference group (Shen and Kogan, 2019). Due to the pervasive existence of labor market segregation, the native-born, relative to their immigrant counterparts, usually possess higher-status occupations associated with higher income (Bosanquet and Doeringer, 1973; Wilson and Portes, 1980; Angrist and Adriana, 2003). Using the British Labor Force Survey, Brynin and Güveli (2012) have demonstrated that due to occupational segregation, there is a significant pay gap in favor of white British workers, who are dominantly native-born, vs. ethnic workers, among whom immigration background is not uncommon. Thus, native-born and immigrant men may hold different starting points, which serve as distinctive baselines in the evaluation of their own economic situations. When unemployed, native-born men evaluate their income loss based on their relatively privileged status in the past and in comparison with their friends, neighbors, and colleagues who remain in their job positions, so as to perceive a deeper drop in their income status. On the contrary, immigrant men are, on average, socioeconomically disadvantaged even when they are employed^1^ (Kogan, 2004, 2011; Brynin and Güveli, 2012). When out of jobs, they are likely to perceive a less strong contrast between their current economic situation and that in the past or that of their friends in similarly disadvantaged job positions. In short, other covariates being equal,

*Hypothesis 2: When being unemployed, native-born men tend to perceive their financial well-being more negatively than immigrant men, which contributes to a larger decline in their life satisfaction, compared to that of their immigrant counterparts*.

In terms of job loss, existing literature has implied the assumption about higher psychological costs among minority groups as compared to the mainstream population (Shields and Wailoo, 2002). We apply this argument to the comparison between immigrant men and their native-born counterparts. First, immigrant men are likely to place particular importance on work, due to their expectations prior to migration and intentions to form new self-identity after migration. Pursuing economic well-being is often the strongest motive for migration, and for the majority of immigrant men, work is the only channel to achieve an economic improvement in the host society (Bartram, 2011). To them, unemployment is not just income loss, but a challenge to their decision of migration. The disillusion of the expectation of improving economic well-being through work in the host society subsequently causes mental harm far more than income loss itself. Second, based on the deprivation approach, immigrant men tend to attach their needs to work more than the native-born, as work is likely to be the foremost arena where the majority of the immigrant population interact with mainstream society, particularly after schooling is completed. For an immigrant man, thus, unemployment is a major disruption of the connection with mainstream society. Very often, an immigrant man's feeling of disconnection from the host society is intertwined with that of frustration due to the disillusion of the original expectation of economic prosperity, subsequently causing a greater degree of distress and decline in life satisfaction that cannot be attributed to income loss alone. Therefore, we hypothesize that other covariates being equal,

*Hypothesis 3: The negative impact of job loss on life satisfaction is greater among immigrant men as compared to native-born men*.

## Comparison Among Immigrant Men Themselves

Needless to say, immigrant men are by no means a homogenous group, which means that their perceptions of unemployment vary. With a focus on the distinction between job loss and income loss, in the present study we mainly discuss the heterogeneity in terms of vulnerability to job loss and income loss, respectively, among immigrant men.

Reasonably, if one's legal status in the host country is tied to employment status, job loss would deprive an immigrant of the legitimacy of residing in the host country. It is thus expected that the more vulnerable an immigrant's status in the host country is to unemployment, the more likely job loss hurts the immigrant for non-economic reasons. On the contrary, the more secure an immigrant's legal status is in the host country, the more similarly he perceives job loss to his native-born counterparts. This is because when one's legal status in the host country is less tied to employment, one can be selective about job options so as to achieve higher income. Upon job loss, therefore, an immigrant with higher socioeconomic status prior to unemployment would suffer more for economic reasons than his counterparts with less bargaining power in the labor market. We therefore hypothesize that job loss should be perceived as more hurtful by immigrant men whose legal status in the host country is more vulnerable to unemployment, whereas income loss would be more hurtful for those whose legal status in the host country is relatively secure. Namely, other covariates being equal,

*Hypothesis 4a: Job loss reduces life satisfaction more for immigrant men whose legal status in the host country depends more on employment status*. And,
*Hypothesis 4b: Income loss reduces life satisfaction more for immigrant men whose legal status in the host country depends less on employment status*.

In terms of income loss, we hypothesize that, similar to the native-born population, less vulnerable immigrant men would also suffer more from subjective financial strain than objective income loss. Thus,

*Hypothesis 4c: A deeper drop in life satisfaction among immigrant men whose legal status is less vulnerable to unemployment is mainly due to perceived financial strain than objective income loss itself*.

## Data, Measurements, and Methods

Data used in this study were drawn from Understanding Society: the UK Household Longitudinal Study (UKHLS) (waves 1–5^2^) between 2009 and 2015 (University of Essex, 2015). The UKHLS incorporates an ethnic minority boost sample, which significantly improves heterogeneity of the immigrant sample concerning countries of origin, migration histories, and other individual characteristics (Knies et al., 2016). We exclusively focused on the active male labor force, aged between 18 and 65, who are either employed, or self-employed, or unemployed but actively seeking employment. Observed individuals include 3,550 immigrant and 16,069 native-born men, with 8,456 and 46,578 individual-wave observations, respectively.

The dependent variable, life satisfaction, refers to an overall assessment of an individual's quality of life according to his or her personal judgment and criteria, and a longer-term state of contentment and well-being (Diener, 1984; Amit, 2010). In the recent development of the SWB literature, life satisfaction has increasingly been used as the proxy of SWB. The measurement of life satisfaction came from a single question in the UKHLS: “Please choose the number which you feel best describes how dissatisfied or satisfied you are with your life overall.” Responses were captured by a seven-point scale ranging from “completely dissatisfied” to “completely satisfied.”

Main independent variables pertain to different aspects of the costs of unemployment. Income loss was measured both objectively and subjectively. In the objective measure, one's position in income distribution, based on household income per capita adjusted by the modified OECD equivalence scale,^3^ was used (coded as 0 = the median 20%, 1 = lowest 20%, 2 = low-median 20%, 3 = median-high 20%, and 4 = the highest 20%). In the subjective measure, perceived financial well-being was captured by one's perceptions of the current and future financial situations. Both measures were coded in the same scale, with the perception of the current financial situation categorized as “just getting by,” “doing all right or well,” and “finding it quite difficult or very difficult,” and the perception of the future financial situation categorized as “about the same,” “better off,” and “worse off.”

Job loss was directly recoded from the “current labor force” in the questionnaire, with being unemployed coded 1 while being paid-employed or self-employed coded 0^4^. We must emphasize that the detrimental effect of unemployment was estimated on the basis of employment status change in two directions—from being employed to unemployed, and from unemployment to reemployment. The majority of existing studies have focused on either of the directions of the employment status change and are unable to address the issue of endogeneity. In terms of the status change into unemployment, individuals with lower levels of life satisfaction are those who have higher risks of being laid off (Leopold et al., 2017). On the contrary, regarding the status change from unemployment to reemployment, individuals with higher levels of life satisfaction tend to be optimistic and proactive in adverse situations, so as to increase their chances of getting reemployed and landing in relatively good positions (McArdle et al., 2007). This means that if self-selection drives estimation biases, it does so in opposite directions for changes from being employed to unemployed and from unemployment to reemployment. Thus, we consider estimating the employment status change in both directions an effective strategy to alleviate the challenge of endogeneity, as estimation biases in opposite directions would more or less cancel each other out at the population level.

Immigrant men were distinguished from native-born men by a dichotomous measure of the immigration status (native born coded 0, including born in England, Wales, Scotland, or Northern Ireland, and non-UK born coded 1, including all other countries). Among immigrants, we further considered the heterogeneity in their vulnerability to unemployment. We first distinguished between recent and established immigrant men. Reasonably, as newcomers, recent immigrant men have a much more vulnerable status in the host country and their self-sustainment is more likely to be tied to employment, compared to their established counterparts. Since this classification among immigrants was not directly available in the questionnaire, we adopted the conventionally used threshold of living in the host country for 10 years to define recent immigrants (duration of residence no more than 10 years, coded 1) and established immigrants (duration of residence more than 10 years, coded 0). This threshold is often used to differentiate between temporary and permanent immigrants across societies. The validity of this measurement has been demonstrated by a recent study about life satisfaction of recent immigrants in Canada (Frank et al., 2016). Whereas the majority of immigrants who plan to leave their countries of residence would do so within 10 years after their first arrival, immigrants who remain in their countries of residence for more than 10 years are more likely to stay permanently (Statistics Canada, 2006; Kone and Sumption, 2019). In a report issued by the Canadian government, established immigrants who live in the country for more than 10 years share similar collective identities with native-born Canadians, while recent immigrants who live in the country for no more than 10 years are significantly less likely to strongly agree with various Canadian identities (Gilkinson and Sauvé, 2010). By utilizing the UKHLS data, we also experimented measuring the duration of residence as either a continuous variable or a categorical variable with a 5-year gap between every two groups. Findings support a significant difference between immigrants residing in the UK for no more than 10 years and those residing in the UK for more than 10 years. Other group differences are negligible. Relevant results are not shown in the paper, but are available upon request.

Among recent immigrants, second, we differentiated immigrants originating from EU countries from those from non-EU countries. During the observational period covered by this study, immigrants with European Economic Area (EEA) nationalities were entitled to the residence right, regardless of their employment status in the UK. This is not the case for non-EU immigrants, whose residence rights are strictly tied to immigration channels through which their entries to the country were initially granted. For those who came for economic, rather than family, reasons, having a job is thus crucial to remain their legal status in the UK. Therefore, we further categorized recent immigrants as recent EU^5^ immigrants and recent non-EU immigrants, based on the original coding of immigrants' countries of origin in the questionnaire^6^.

Other individual characteristics that have commonly been examined as factors influencing how one feels about unemployment were controlled, including age and its quadratic form (due to a non-linear relationship shown by existing literature as aforementioned), having a religion (yes = 1; no = 0), marital status in combination with the partner's employment status (single = 0, never married = 1; having a partner who is not unemployed = 2; having an unemployed partner = 3; widowed and divorced = 4), educational qualification, physical well-being, household composition, and access to unemployment benefits. Educational qualification was measured by six dummy categories: having a degree, having other degrees, A-level, GCSE, other qualifications, and no qualification, with the group of “no qualification” used as a reference group. Physical well-being was measured by a score between 0 and 100, calculated based on a series of self-reported questions on health issues and physical activities^7^. Type of household composition included eight categories: a working couple without any child (used as a reference group), a one-person household, a lone-parent household, a senior couple (referring to couples with at least one side retired) without any child, a couple with one child, a couple with two children, a couple with three children, and others. The variable “unemployment benefits” was measured by a dichotomous measure with “getting any kind(s) of unemployment benefit(s)” coded 1 and “not getting any” coded 0^8^. Descriptive statistics are shown as the Appendix.

Analyses were carried out by using fixed-effect modeling. Subjective measures such as life satisfaction are often faced with challenges of endogeneity. For example, individuals with optimistic personalities may view the unemployment experience more positively than those who are more pessimistic. The personality difference would consequently cause a smaller estimated effect of unemployment for optimistic individuals, whereas a larger one for pessimistic individuals. Such issues would not exist in fixed-effect modeling. By estimating only within-individual variations, the fixed effect model can effectively address unobserved, individual-specific, and time-invariant disturbances. In all models about the immigrant population, standard errors were estimated by using the countries of origin as the cluster variable, with the consideration that the shape of the distribution of each independent variable may be country-specific across immigrants. The model specification is: *y*_*it*_ = *x*′_*it*_ β + ε_*it*_, where *i* = 1, …, *n* (individuals), *t* = 1, …, *T* (waves), and *x*′_*it*_ β = β_0_ + β_1_*x*_*it*,1_ + … + β_*K*_*x*_*it*,*K*_ (Rabe-Hesketh and Skrondal, 2008). The dependent variable “life satisfaction” was treated as a continuous variable. In their methodological comparison, Ferrer-i-Carbonell and Frijters (2004) have shown that assuming ordinality or cardinality of SWB (such as happiness) scores did not make significant differences in estimations on the changes in satisfaction and the corresponding standard errors. Under this condition, more parsimonious estimations by using life satisfaction as a continuous variable were preferred.

## Differences in Perceptions of Unemployment Between Immigrant and Native-Born Men

In Table 1, Models 1 through 4 present fixed-effect estimations for the whole sample of men in the labor force. We first estimated the coefficient of unemployment, without controlling for any financial measures. We subsequently controlled for objective income status and subjective financial well-being, separately and together, to observe the extent to which the coefficient of unemployment can be reduced by taking into account objective and subjective measures of income loss. Covariates were controlled in all models.

**Table 1:** Fixed-effect estimations on men's life satisfaction by job loss and income loss, the United Kingdom, 20019–2015.

	**Model 1**	**Model 2**	**Model 3**	**Model 4**
Unemployment	−0.250^***^	−0.227^***^	−0.154^***^	−0.142^***^
	(0.015)	(0.017)	(0.014)	(0.014)
**Objective income (references** **=** **median 20%)**				
Bottom 20%		−0.107^***^		−0.067^***^
		(0.012)		(0.009)
Lower-middle 20%		−0.007		0.005
		(0.005)		(0.005)
Upper-middle 20%		0.017^***^		0.004
		(0.004)		(0.004)
Upper 20%		0.029^**^		0.013
		(0.009)		(0.009)
**Subjective financial well-being (reference** **=** **neutral)**				
Better off_current			0.211^***^	0.208^***^
			(0.008)	(0.008)
Worse off_current			−0.388^***^	−0.384^***^
			(0.007)	(0.007)
Better off_future			0.033^***^	0.033^***^
			(0.008)	(0.008)
Worse off_future			−0.064^***^	−0.065^***^
			(0.003)	(0.003)
**Interactions with immigration status**				
Unemployment	−0.024	−0.048	−0.103^+^	−0.117^*^
	(0.066)	(0.064)	(0.051)	(0.049)
Bottom 20%		0.163^*^		0.131
		(0.076)		(0.077)
Lower-middle 20%		0.058		0.045
		(0.055)		(0.061)
Upper-middle 20%		0.068		0.071
		(0.068)		(0.062)
Upper 20%		0.086		0.092
		(0.095)		(0.095)
Better off_current			−0.045	−0.045
			(0.043)	(0.042)
Worse off_current			0.214^**^	0.209^**^
			(0.074)	(0.073)
Better off_future			0.045	0.044
			(0.057)	(0.057)
Worse off_future			−0.081	−0.082
			(0.070)	(0.071)
Constant	4.360^***^			4.370^***^
	(1.155)			(1.090)
Cases	55,079			54,362
Individuals	19,642			19,515
**Variance components**				
Level 2 S.D. (σ_u)	1.376			1.325
Level 1 residual S.D. (σ_e)	1.106			1.096
Intraclass corr. (ρ)	0.608			0.594

*Understanding Society: the UKHLS, 2009–2015 (University of Essex, 2015). The following covariates were controlled in all models: Age, age squared, having a religion, marital status, education, physical well-being, status in the labor force, and type of household composition. Duration of residence in the UK was controlled for immigrants. Robust standard errors in parentheses*.

*** p < 0.001

** *p < 0.01*,

* *p < 0.05*,

+ *p < 0.1*.

Model 1 shows that without controlling for income loss, life satisfaction of unemployed men is 0.25 points lower than that of employed men, and this detrimental effect of unemployment on life satisfaction does not significantly differ by immigration status, as shown by the non-significant interaction term. Model 2 includes objective income status and its interaction with immigration status. Compared to Model 1, controlling for objective income status slightly reduces the detrimental effect of unemployment on life satisfaction, from 0.25 to 0.23 points. The coefficient of each income status refers to the effect of income status change, because fixed-effect modeling only estimates over-time changes occurring on each individual, namely, within-individual variations. For example, the coefficient of “bottom 20%” means that comparing to those moving to the median 20%, those who have dropped to the bottom 20% report life satisfaction by 0.107 points lower, whereas those who have moved to the upper-middle and upper tiers report life satisfaction by 0.017 and 0.029 points higher, respectively, other covariates being equal. Interaction terms show that the immigrant-native gap in life satisfaction is significant only among those who have dropped to the bottom of the income distribution, with immigrants being 0.163-point more satisfied. In other words, native-born men suffer more than immigrant men from an adverse income status change.

In Model 3, perceived financial well-being is included, which greatly reduces the detrimental effect of unemployment on life satisfaction, from 0.25 to 0.15 points. This negative impact of unemployment differs by immigration status, though with marginal significance. The impact of perceived financial well-being on life satisfaction varies between immigrant and native-born men significantly. Other covariates being equal, positive perceptions of one's financial situation boost whereas negative perceptions hinder life satisfaction, with perceptions of the current situation playing a greater role than those of the future situation. Interaction terms show that for those who hold negative perceptions of the current financial situation, immigrant men report higher life satisfaction than their native-born counterparts by 0.214 points. Namely, perceived current financial hardship hurts life satisfaction of native-born men more than that of immigrant men.

Model 4 is the full model with job loss as well as both objective and subjective measures of income loss taken into account. Other covariates being equal: unemployment reduces life satisfaction—for native-born men—by 0.14 points, and it further reduces life satisfaction of unemployed immigrant men by additional 0.12 points. When income loss is measured by both objective and subjective terms, one can see that the effects of objective income status become less salient—in terms of statistical significance and magnitudes of coefficients, compared to corresponding coefficients in Model 2. The effects of subjective financial well-being remain by and large similar to those in Model 3. While the difference in objective income loss is no longer significant between immigrant and native-born men, the subjective perception of income loss, indicated by perceiving the current situation being worse off, is still significant, with immigrant men feeling more positive than their native-born counterparts.

To summarize, findings in Models 1 through 4 show that: (1) unemployment indeed has a detrimental impact on the level of men's life satisfaction; (2) a part of the detrimental effect of unemployment is attributed to pecuniary reasons; (3) perceived financial strain or hardship plays a more important role than objective income loss in affecting unemployed men's life satisfaction; and (4) with both objective and subjective measures of income loss taken into account, jobs loss by itself hurts life satisfaction more for immigrant men than their native-born counterparts. Namely, Hypotheses 2 and 3 are supported. Hypothesis 1 is supported only when perceived financial well-being is not taken into account.

To gain a further understanding about how perceptions of unemployment and the resulting consequences on life satisfaction differ by immigration status, we subsequently ran separate models for immigrant and native-born men as presented by Table 2. By comparing coefficients of unemployment in Models 5 through 8 and Models 9 through 12, it is clear that unemployment has a generally larger negative impact on immigrant men's life satisfaction than native-born men's. Moreover, while the inclusion of objective and subjective financial measures does not reduce the negative effect of unemployment considerably for immigrant men, it does so for native-born men. Comparing the full models (Models 8 and 12), one can see that the effect of unemployment (job loss) is much larger for immigrant than native-born men (−0.32 vs. −0.13). In terms of pecuniary costs, objective income status has no significant impact on immigrant men's life satisfaction, and has only a slight effect on life satisfaction of native-born men at the bottom 20 percent of the income hierarchy (relative to their counterparts at the middle 20 percent). Subjective income loss—one's perception of being worse off, particularly about the current situation—presents a much larger negative impact on life satisfaction among native-born men relative to immigrant men (−0.38 vs. −0.18).

**Table 2:** Fixed-effect estimations on life satisfaction by job loss and income loss for immigrant and native-born men, the United Kingdom, 2009–2015.

	**Immigrant men**				**Native-born men**			
	**Model 5**	**Model 6**	**Model 7**	**Model 8**	**Model 9**	**Model 10**	**Model 11**	**Model 12**
Unemployment	−0.335^***^	−0.335^***^	−0.315^***^	−0.316^***^	−0.237^***^	−0.215^***^	−0.142^***^	−0.130^***^
	(0.060)	(0.059)	(0.051)	(0.051)	(0.035)	(0.036)	(0.036)	(0.036)
**Objective income (references** **=** **median 20%)**								
Bottom 20%		0.057		0.063		−0.106^***^		−0.066^*^
		(0.074)		(0.075)		(0.027)		(0.027)
Lower-middle 20%		0.056		0.053		−0.007		0.005
		(0.055)		(0.061)		(0.022)		(0.022)
Upper-middle 20%		0.082		0.071		0.017		0.004
		(0.066)		(0.060)		(0.022)		(0.022)
Upper 20%		0.118		0.106		0.029		0.013
		(0.093)		(0.095)		(0.027)		(0.027)
**Subjective financial well-being (references** **=** **neutral)**								
Better off_current			0.159^***^	0.157^***^			0.211^***^	0.209^***^
			(0.040)	(0.039)			(0.019)	(0.019)
Worse off_current			−0.174^*^	−0.175^*^			−0.387^***^	−0.383^***^
			(0.072)	(0.070)			(0.026)	(0.026)
Better off_future			0.075	0.073			0.034^*^	0.034^*^
			(0.058)	(0.058)			(0.017)	(0.017)
Worse off_future			−0.139^+^	−0.142^+^			−0.065^**^	−0.066^**^
			(0.070)	(0.070)			(0.020)	(0.020)
Constant	1.687	1.65	1.921	1.879	4.645^**^	4.658^**^	4.720^***^	4.731^***^
	(2.542)	(2.586)	(2.720)	(2.763)	(1.438)	(1.438)	(1.433)	(1.433)
Cases	8,456	8,452	8,292	8,288	46,578	46,567	46,040	46,029
Individuals	3,550	3,548	3,503	3,501	16,069	16,066	15,994	15,991
**Variance components|**								
Level 2 S.D. (σ_u)	2.205	2.194	2.093	2.084	1.352	1.354	1.282	1.284
Level 1 residual S.D. (σ_e)	1.221	1.222	1.216	1.217	1.086	1.085	1.076	1.076
Intraclass corr. (ρ)	0.765	0.763	0.747	0.746	0.608	0.609	0.587	0.588

*Understanding Society: the UKHLS, 2009–2015 (University of Essex, 2015). The following covariates were controlled in all models: age, age squared, having a religion, marital status, education, physical well-being, status in the labor force, and type of household composition. Duration of residence in the UK was controlled for immigrants. Robust standard errors in parentheses*.

*** p < 0.001

** *p < 0.01*,

* *p < 0.05*,

+ *p < 0.1*.

**Figure 1:**
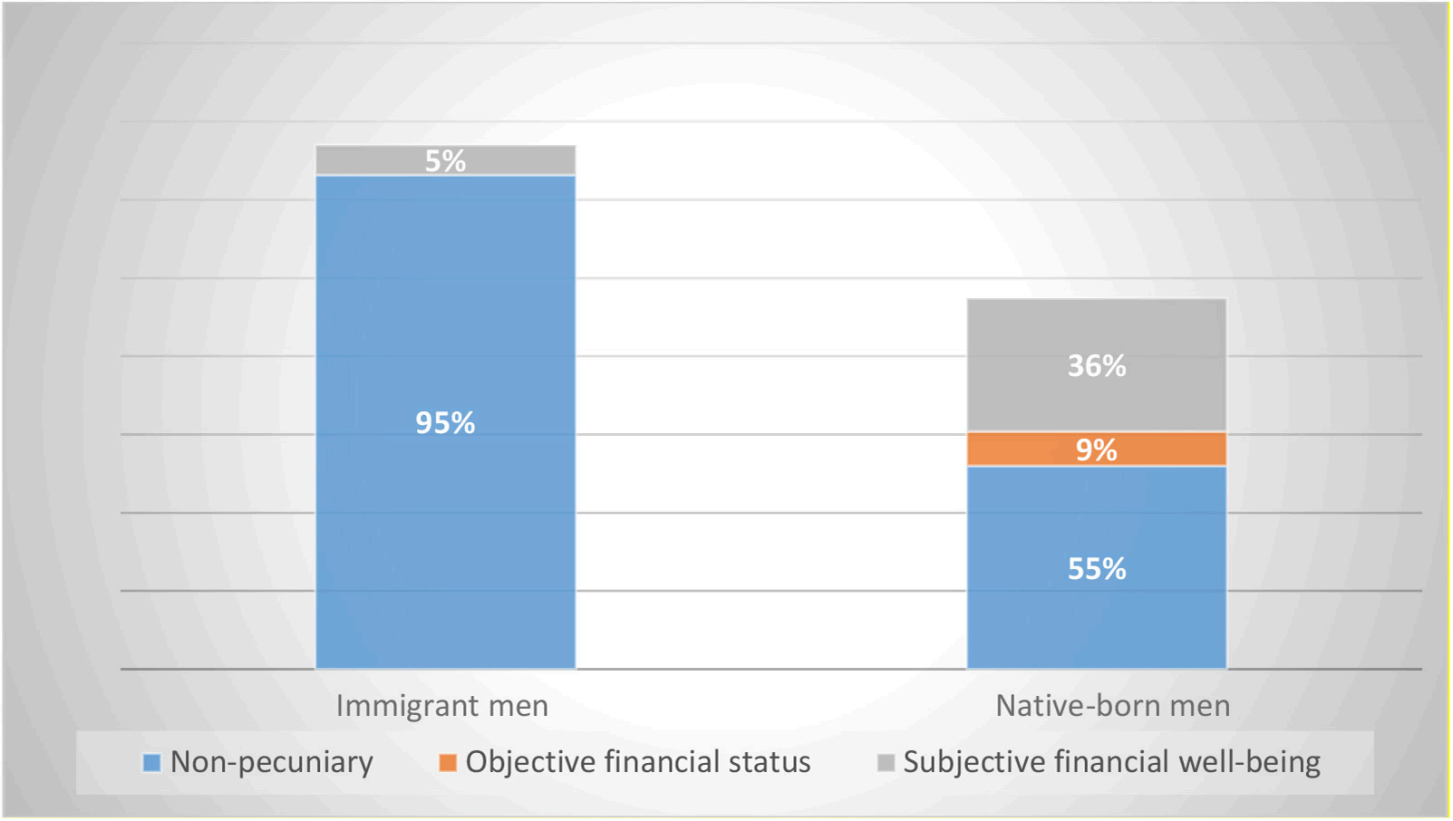
Composition of the detrimental effect of unemployment on life satisfaction for immigrant and native-born men. Authors' own calculations based on Table 1. Data source: Understanding Society: the UKHLS, 2009–2015 (University of Essex, 2015).

We further calculate the composition of the detrimental effect of unemployment, based on estimations from Table 2. As shown by Figure 1, for immigrant men, 95 percent of the detrimental effect of unemployment is non-pecuniary, namely, due to job loss *per se*, and subjective financial strain explains the remaining 5 percent. For native-born men, by contrast, only 55 percent of the negative impact of unemployment is due to non-pecuniary costs, whereas 45 percent is pecuniary, in which the contribution of subjective income loss—perceived financial strain—is 4 times as large as that of objective income loss. In short, the detrimental effect of job loss is higher for immigrant men compared to native-born men. This is in contrast with the higher detrimental effect of income loss for native-born men, which is mainly due to a larger negative impact of perceived financial strain among native-born men compared to immigrant men.

## Differences in Perceptions of Unemployment Among Immigrant Men Themselves

In this section, we narrow down the analysis to the immigrant subsample. Table 3 presents the same modeling strategies used in the previous two tables with a focus on the distinction between recent and established immigrants. From Models 13 through 16, unemployment significantly reduces life satisfaction for all immigrant men in the subsample, but more so for recent immigrants, as shown by the significantly negative interaction coefficients between unemployment and the recent immigrant status. The full model (Model 16) shows that other covariates being equal: recent immigrant men are generally more satisfied with their lives than their established counterparts (coef. = 0.17). This finding is consistent with existing literature about the declining trend of life satisfaction among immigrants, as the duration of residence in the host country increases and across generations (Safi, 2010; Bartram, 2011). However, once unemployed, recent immigrant men's life satisfaction suffers more than established immigrants' due to job loss, as the negative impact of unemployment is 0.34 points larger for recent immigrants compared to established immigrants.

**Table 3:** Fixed-effect estimations on life satisfaction by job loss and income loss between recent and established immigrant men, the United Kingdom, 2009–2015.

	**Model 13**	**Model 14**	**Model 15**	**Model 16**
Unemployment	−0.213^***^	−0.201^***^	−0.194^***^	−0.184^***^
	(0.054)	(0.050)	(0.039)	(0.040)
Recent immigrants	0.148	0.073	0.228^+^	0.165^*^
	(0.112)	(0.091)	(0.112)	(0.072)
Unem^*^recent immigrants	−0.309^*^	−0.344^*^	−0.313^*^	−0.343^*^
	(0.134)	(0.151)	(0.124)	(0.139)
**Objective income (reference** **=** **median 20%)**				
Bottom 20%		−0.016		−0.005
		(0.103)		(0.104)
Lower-middle 20%		0.041		0.036
		(0.059)		(0.069)
Upper-middle 20%		0.026		0.014
		(0.077)		(0.072)
Upper 20%		0.114		0.115
		(0.099)		(0.101)
**Subjective financial well-being (references** **=** **neutral)**				
Better off_current			0.203^**^	0.200^**^
			(0.059)	(0.061)
Worse off_current			−0.118	−0.112
			(0.091)	(0.088)
Better off_future			0.070	0.070
			(0.052)	(0.052)
Worse off_future			−0.213^*^	−0.217^**^
			(0.077)	(0.077)
**Interactions of income loss and recent immigrant men**				
Bottom 20%		0.186		0.169
		(0.190)		(0.198)
Lower-middle 20%		0.033		0.032
		(0.100)		(0.114)
Upper-middle 20%		0.156^+^		0.163^+^
		(0.082)		(0.083)
Upper 20%		0.004		−0.039
		(0.130)		(0.142)
Better off_current			−0.099	−0.096
			(0.091)	(0.102)
Worse off_current			−0.155	−0.170
			(0.151)	(0.148)
Better off_future			0.007	0.010
			(0.057)	(0.057)
Worse off_future			0.265^*^	0.276^*^
			(0.123)	(0.123)
Constant	2.927	2.724	2.959	2.755
	(2.626)	(2.712)	(2.691)	(2.787)
Cases	8,501	8,497	8,337	8,333
Individuals	3,573	3,571	3,526	3,524
**Variance components**				
Level 2 S.D. (σ_u)	1.416	1.414	1.399	1.398
Level 1 residual S.D. (σ_e)	1.222	1.222	1.217	1.217
Intraclass corr. (ρ)	0.573	0.572	0.570	0.569

*Understanding Society: the UKHLS, 2009–2015 (University of Essex, 2015). The following covariates were controlled in all models: age, age squared, having a religion, marital status, education, physical well-being, status in the labor force, type of household composition and duration of residence in the UK. Robust standard errors in parentheses*.

*** p < 0.001

** *p < 0.01*,

* *p < 0.05*,

+ *p < 0.1*.

In terms of income loss, an anticipation of future financial hardship decreases established immigrant men's life satisfaction by 0.22 points, compared to established immigrant men who foresee no financial change in the future. However, it does not seem to decrease recent immigrants' life satisfaction. More precisely, even with the perception of future financial hardship, recent immigrants' life satisfaction is still 0.06 points (= 0.28–0.22) higher than established immigrants who anticipate no financial change in the future. Due to a trivial role objective income loss plays in explaining unemployment costs among the employed immigrant men as shown by previous two tables, the difference between recent and established immigrants is negligible. Therefore, unemployed established immigrant men bear higher psychological costs of income loss than unemployed recent counterparts, due to their stronger perception of future financial strain. Meanwhile, we find strong evidence to support a significantly larger negative impact of job loss on life satisfaction for recent immigrant men, compared to established immigrant men.

In Table 4, we further differentiate recent immigrant men by the EU status of their countries of origin and report results in comparison with those shown by Table 3. By comparing Models 13 and 17, one can see that without controlling for measures of income loss, coefficients of unemployment are similar, and that a greater decline in life satisfaction of unemployed recent immigrant men mainly exists among those from non-EU countries. While Model 14 reports non-significant coefficients of objective income status change, Model 18 shows that when income drops to the bottom 20% of the distribution, recent EU immigrant men perceive this change more positively than their established counterparts (coef. = 0.41). Model 19 shows that higher life satisfaction of recent immigrant men with the perception of future financial hardship is mainly driven by the positive attitude held by those from non-EU countries. Perceived financial hardship hurts life satisfaction of recent EU immigrant men significantly more than that of their established counterparts, whether in terms of the current or future situation. The full model (Model 20) confirms all the above findings.

**Table 4:** Fixed-effect estimations on life satisfaction by job loss and income loss among recent EU, recent non-EU and established immigrant men, the United Kingdom, 2009–2015.

	**Model 17**	**Model 18**	**Model 19**	**Model 20**
Unemployment	−0.216^***^	−0.206^***^	−0.203^***^	−0.195^***^
	(0.056)	(0.053)	(0.041)	(0.043)
**Reference: established immigrant men**				
Recent EU	0.016	−0.066	0.306	0.281
	(0.405)	(0.493)	(0.351)	(0.419)
Recent non-EU	0.164	0.099	0.192	0.133^*^
	(0.118)	(0.087)	(0.128)	(0.063)
Unem^*^recent EU	0.009	−0.102	0.007	−0.061
	(0.488)	(0.467)	(0.399)	(0.430)
Unem^*^recent non-EU	−0.331^*^	−0.359^*^	−0.318^*^	−0.345^*^
	(0.130)	(0.151)	(0.127)	(0.145)
Bottom 20%		−0.013		−0.002
		(0.104)		(0.105)
Lower-middle 20%		0.045		0.041
		(0.059)		(0.069)
Upper-middle 20%		0.021		0.011
		(0.077)		(0.073)
Upper 20%		0.113		0.115
		(0.100)		(0.102)
Better off_current			0.183^**^	0.182^**^
			(0.055)	(0.057)
Worse off_current			−0.122	−0.118
			(0.087)	(0.084)
Better off_future			0.074	0.073
			(0.052)	(0.051)
Worse off_future			−0.215^*^	−0.218^**^
			(0.077)	(0.077)
**Interactions of income loss and recent eu/non-eu immigrant men (references: established)**				
Bottom 20%^*^recent EU		0.412^*^		0.417^+^
		(0.189)		(0.241)
Bottom 20%^*^recent non-EU		0.155		0.147
		(0.210)		(0.218)
Lower-middle 20%^*^recent EU		0.003		−0.033
		(0.236)		(0.229)
Lower-middle 20%^*^recent non-EU		0.030		0.027
		(0.101)		(0.115)
Upper-middle 20%^*^recent EU		0.270		0.372
		(0.262)		(0.244)
Upper-middle 20%^*^recent non-EU		0.140		0.144
		(0.091)		(0.094)
Upper 20%^*^recent EU		−0.036		−0.106
		(0.273)		(0.236)
Upper 20%^*^recent non-EU		0.006		−0.032
		(0.143)		(0.161)
Better off_current^*^recent EU			−0.223^+^	−0.292^*^
			(0.126)	(0.114)
Better off_current^*^recent non-EU			−0.059	−0.055
			(0.093)	(0.103)
Worse off_current^*^recent EU			−0.678^*^	−0.758^**^
			(0.251)	(0.261)
Worse off_current^*^recent non-EU			−0.088	−0.100
			(0.161)	(0.157)
Better off_future^*^recent EU			−0.069	−0.074
			(0.205)	(0.198)
Better off_future^*^recent non-EU			0.016	0.020
			(0.064)	(0.066)
Worse off_future^*^recent EU			−0.431^*^	−0.455^*^
			(0.165)	(0.169)
Worse off_future^*^recent non-EU			0.387^***^	0.398^***^
			(0.077)	(0.079)
Constant	1.295	1.124	1.364	1.189
	(2.655)	(2.743)	(2.819)	(2.912)
Cases	8,456	8,452	8,292	8,288
Individuals	3,550	3,548	3,503	3,501
**Variance components**				
Level 2 S.D. (σ_u)	2.284	2.253	2.204	2.169
Level 1 residual S.D. (σ_e)	1.221	1.222	1.215	1.216
Intraclass corr. (ρ)	0.778	0.773	0.767	0.761

*Understanding Society: the UKHLS, 2009–2015 (University of Essex, 2015). The following covariates were controlled in all models: age, age squared, having a religion, marital status, education, physical well-being, status in the labor force, type of household composition and duration of residence in the UK. Robust standard errors in parentheses*.

*** p < 0.001

** *p < 0.01*,

* *p < 0.05*,

+ *p < 0.1*.

Overall, findings from Tables 3, 4 show that job loss reduces life satisfaction more for recent than established immigrant men, and this gap mainly exists between recent immigrant men from non-EU countries and their established counterparts. Perceived financial well-being plays a bigger role than objective income status change in life satisfaction of all immigrant men. As shown by the final model (Model 20), a positive perception of the current financial situation increases, whereas a negative perception of the future financial situation decreases, life satisfaction of established immigrant men. Recent immigrant men show a higher level of life satisfaction than established counterparts when perceiving future financial hardship, but this is solely driven by the pattern observed among those from non-EU countries. By contrast, recent EU immigrant men feel significantly unsatisfied, and their life satisfaction drops much further compared to that of the established counterparts, when they perceive either current or future financial hardship.

In short, our results confirm that job loss leads to higher psychological costs for those whose legal status in the host country depends more on employment status, i.e., recent non-EU immigrant men. Hypothesis 4a is supported. We also find that compared to recent non-EU immigrants, established immigrant men bear higher psychological costs of perceived income loss, indicated by the perception of future financial hardship. When comparing established immigrant men with their recent EU counterparts, one can observe significantly lower levels of life satisfaction among the latter group, particularly with perceptions of financial hardship. As shown in the descriptive statistics (Appendix), the majority of established immigrant men originate from non-EU countries. This means that by sharing equal rights of employment and residence with the native-born, recent EU immigrant men may possess a legal status even less vulnerable to unemployment. The comparison between established and recent EU immigrant men further confirms that when an immigrant's legal status in the host country is less contingent on employment, individuals would put more emphasis on the pecuniary aspect of work and consequently feel more stressed when perceiving financial hardship. Namely, Hypotheses 4b and 4c are supported.

## Conclusions and Discussion

Existing literature about the detrimental effect of unemployment on life satisfaction has mainly been centered on the debate about the relative importance of job loss and income loss. By drawing data from a countrywide longitudinal dataset in the UK, this study provides new evidence to the debate. Moreover, this study contributes to the literature by examining to what extent men's immigration status moderates the effects of unemployment on life satisfaction.

Our findings confirm that for native-born men, both job loss and income loss play significant roles in the decline of life satisfaction, and that the detrimental effect of income loss is mainly due to perceived financial strain, rather than objective income loss. Among immigrant men, job loss by itself is the dominant reason for the decline in life satisfaction during unemployment. Only a small proportion of the detrimental effect of unemployment is pecuniary, and this proportion can only be explained by subjective perceptions of financial strain rather than objective income loss. Our results also show that the total detrimental effect of unemployment on life satisfaction is much larger for immigrant than native-born men, and this is mainly due to the greater negative impact of job loss, rather than income loss.

The above findings suggest that immigrant and native-born men perceive unemployment differently. While native-born men consider work primarily a means of economic independence, immigrant men gain greater life satisfaction from the non-pecuniary aspect of work. We speculate that this is because native-born men's self-evaluation about their position in the society is more vulnerable to income loss, whereas the legal status of immigrant men in the country is more vulnerable to job loss. Namely, the more secure one's legal status is in the society, the more likely one would emphasize the pecuniary aspect over the non-pecuniary aspect of work. Our further investigation within the group of immigrant men has confirmed this speculation. With a focus on the extent to which an immigrant man's legal status in the host country is vulnerable to unemployment, we distinguished between established and recent immigrant men, and for the latter group, we made a further distinction between recent EU and non-EU immigrant men. Findings show that job loss causes a deeper decline in life satisfaction for those whose legal status in the host country depends more on employment status, i.e., recent non-EU immigrants, whereas income loss causes a deeper decline in life satisfaction for those whose residence right in the host country is not or less attached to employment, i.e., recent EU and established immigrants.

Above all, comparisons between immigrant and native-born men and among immigrant men themselves reflect a similar pattern: People whose residence right in the society is attached to employment emphasize more on the non-pecuniary aspect of work and thus suffer more from job loss. On the contrary, those whose residence right is less attached to employment emphasize more on the pecuniary aspect of work and thus suffer more from income loss accompanying unemployment. This may be because people with vulnerable status in the society, i.e., recent non-EU immigrants, are likely to consider work the foremost channel to build social connections and to avoid isolation in the host country. Others, including native-born men, established and recent EU immigrant men, are likely to consider work the dominant channel of upward mobility in the socioeconomic hierarchy in mainstream society. This divergence may fundamentally be driven by the assimilation argument, with recent non-EU immigrant men being less assimilated whereas recent EU and established immigrant men being more assimilated into the norms of mainstream society. Empirical demonstration of the assimilation argument is beyond the scope of the present study and should be explored in future research.

Another possible explanation is self-selection. Immigrants moving for economic reasons are often driven by their ambitions and motivations to achieve better economic lives. Compared to their counterparts staying in their countries of origin, economic immigrants are likely to be able to take higher risks for greater career success. The self-selection argument may well-explain why unemployed immigrants suffer more from job loss *per se*. However, it cannot explain why recent EU and non-EU immigrant men perceive and feel about unemployment differently, particularly because the portion of economic immigrants is larger within the EU than non-EU group (Vargas-Silva and Rienzo, 2019). It is possible that the immigration screening process applied to immigrants from non-EU countries drives a much stronger positive self-selection mechanism. For one, economic immigrants from non-EU countries could be more career-driven than their counterparts from EU countries, so as to make extra efforts to go through the immigration procedure. For the other, people who manage to move to the UK from non-EU countries are likely to be the advantaged in their countries of origin. For example, our results show that when employed, recent non-EU immigrant men report a higher level of life satisfaction than native-born men, while the level of life satisfaction does not significantly differ among native-born men, recent EU and established immigrant men. Future research is thus faced with the challenge of estimating immigrants' perceptions of and evaluations about unemployment with different extents of self-selection among various immigrant groups taken into account.

It is necessary to restate the exclusion of women in this study. Women's labor force participation is a multi-faceted phenomenon, due to their reproductive roles and family obligations. Great variations in perceptions of labor market participation exist among women. By contrast, men's participation in the labor market is a relatively universal phenomenon and their perceptions of unemployment are much less heterogeneous compared to women's, as the social expectation of men being providers is very much consistent across societies (Cohn, 1978). For this reason, factors causing heterogeneity in women's perceptions of unemployment would be less significant in the men subsample. This naturally calls for a new task in future research, which is to carry out an analysis of women in the labor force to complete the picture of the impact of unemployment on life satisfaction. Women's subjective reactions to unemployment are expected to be significantly different from men's. The inclusion of immigration status would further complicate the scenario. Therefore, proper strategies that can capture factors of the heterogeneity of women's—particularly immigrant women's—perceptions of work as well as feelings about unemployment will make significant contributions to the literature.

Future work notwithstanding, this paper is one of the very few studies analyzing the effect of unemployment on the immigrant men. We find that in addition to individual characteristics discussed in existing literature, investigations about the negative impact of unemployment on life satisfaction should take into account immigration status in general and the extent to which one's legal status in the society is vulnerable to unemployment in particular.

## Data Availability Statement

The datasets generated for this study are available on request to the corresponding author.

## Author's Note

The authors confirm that this study is original research. It has not been previously published or been under consideration for publication elsewhere, either in whole or in part.

## Author Contributions

JS has completed the data analyses, drafted the complete paper and revised the paper based upon the IK's comments. IK has supervised the process of data analyses, reviewed, and commented on various versions of the paper.

### Conflict of Interest

The authors declare that the research was conducted in the absence of any commercial or financial relationships that could be construed as a potential conflict of interest.

## Footnotes

^1^It is true that highly educated and highly skilled immigrants have much better chances to be employed in high-status positions in the host-country labor market. However, this only constitutes a small portion of the immigrant population. Furthermore, studies show that the immigrant-native wage gap is wider, rather than narrower, at the higher end of the income distribution (Chiswick and Miller, 2008; Dell'Aringa et al., 2015).

^2^Wave 6 was not available during the completion of data analysis for this study.

^3^For details, please refer to the variable “ieqmoedc_dv”—Modified OECD equivalence scale in UKHLS Codebook. Available at: https://www.understandingsociety.ac.uk/documentation/mainstage/dataset-documentation.

^4^The original variable in the questionnaire “current labor force status” includes the following categories: (1) self-employed, (2) paid employment (either full time or part time), (3) unemployed, (4) retired, (5) on maternity leave, (6) family care or home, (7) full-time student, (8) long-term sick, or disabled, (9) governmental training scheme, (10) unpaid family business, (11) on apprenticeship and (12) doing something else. Category (3) does not distinguish between short-term and long-term unemployment. Respondents inactive in the labor market, namely, categories (4–12) were excluded from the analysis.

^5^There is no country that is an EEA country but does not belong to the EU in the original coding of immigrants' source countries in the questionnaire. For convenience, we thus equated EEA countries to EU countries in this study.

^6^Categories of immigration, e.g., economic immigrants and those immigrating for family reasons, were not distinguished in the original UKHLS data. However, our exclusive focus on men in the labor force in this study has significantly alleviated this limitation. Men's labor market participation is consistently higher than women's in UK society, and this gender gap is even more salient among immigrants (Office for National Statistics, 2019). Namely, an immigrant man has a much greater chance than an immigrant women to actively participate in the labor market, regardless of the channel of his entry into the UK.

^7^More details can be seen in the UKHLS Codebook, University of Essex 2015.

^8^For those who moved from employment into unemployment, this variable would not vary, as no unemployment benefits would apply to employed individuals. This variable was meaningful only for those who moved from unemployment to reemployment. Compared to results presented in the main text, results without controlling for unemployment benefits show a much more consistent level of life satisfaction across waves for each respondent (indicated by the much larger interclass correlation ρ), regardless of the employment status change (results are not presented in the paper but are available upon request). That is to say, having unemployment benefits or not indeed made a difference in one's perception of being unemployed and the subsequent level of life satisfaction. We therefore took into account this variable, despite the fact that it was meaningful only for one direction of the employment status change.
