# Supplementary material for: Job Loss or Income Loss: How the Detrimental Effect of Unemployment on Men's Life Satisfaction Differs by Immigration Status
Source: Front Sociol. 2020 Feb 28;5:10. doi: 10.3389/fsoc.2020.00010 (PMC8022760; doi:10.3389/fsoc.2020.00010)
Supplement: Supplementary file 1 [file Table_1.pdf]

Appendix. Descriptive Statistics of Used Variables, Understanding Society, the United Kingdom, 2009-2015

	Immigrant men (N=16,069 / n=3550)					Native-born men (N=46,578 / n=8,456)			
Groups of immigrants	Overall %	Between %	Within %						
Established	61.88	61.10	94.39						
Established EU-origin	10.35	9.83	100.00						
Established non-EU-origin	89.65	90.17	100.00						
Recent EU	5.92	6.18	95.26						
Recent non-EU	32.21	40.82	90.76						
**Variables**		**Mean**	**Std. Dev.**	**Min**	**Max**	**Mean**	**Std. Dev.**	**Min**	**Max**
Life satisfaction	overall	4.962	1.496	1.000	7.000	5.074	1.473	1.000	7.000
	between		1.282	1.000	7.000		1.269	1.000	7.000
	within		0.948	0.162	9.212		0.890	0.274	9.574
Unemployment	overall	0.104	0.305	0.000	1.000	0.083	0.276	0.000	1.000
	between		0.271	0.000	1.000		0.249	0.000	1.000
	within		0.185	-0.696	0.904		0.168	-0.717	0.883
Objective income status									
Bottom 20%	overall	0.280	0.449	0.000	1.000	0.170	0.375	0.000	1.000
	between		0.399	0.000	1.000		0.330	0.000	1.000
	within		0.276	-0.520	1.080		0.237	-0.630	0.970
Lower-middle 20%	overall	0.217	0.412	0.000	1.000	0.185	0.389	0.000	1.000
	between		0.319	0.000	1.000		0.303	0.000	1.000
	within		0.302	-0.583	1.017		0.280	-0.615	0.985
Middle 20%	overall	0.179	0.383	0.000	1.000	0.202	0.402	0.000	1.000
	between		0.293	0.000	1.000		0.302	0.000	1.000
	within		0.280	-0.621	0.979		0.298	-0.598	1.002
Upper-middle 20%	overall	0.158	0.364	0.000	1.000	0.218	0.413	0.000	1.000
	between		0.282	0.000	1.000		0.313	0.000	1.000
	within		0.259	-0.642	0.958		0.296	-0.582	1.018
Upper 20%	overall	0.166	0.373	0.000	1.000	0.225	0.418	0.000	1.000
	between		0.319	0.000	1.000		0.352	0.000	1.000
	within		0.206	-0.634	0.966		0.238	-0.575	1.025
Subjective financial well-being									
Worse off_current	overall	0.467	0.499	0.000	1.000	0.605	0.489	0.000	1.000
	between		0.441	0.000	1.000		0.425	0.000	1.000
	within		0.279	-0.333	1.267		0.279	-0.195	1.405
Getting by_currrent	overall	0.306	0.461	0.000	1.000	0.274	0.446	0.000	1.000
	between		0.373	0.000	1.000		0.356	0.000	1.000

	within		0.319	-0.494	1.106		0.309	-0.526	1.074
Better off_current	overall	0.227	0.419	0.000	1.000	0.121	0.326	0.000	1.000
	between		0.363	0.000	1.000		0.275	0.000	1.000
	within		0.252	-0.573	1.027		0.210	-0.679	0.921
Worse off_future	overall	0.367	0.482	0.000	1.000	0.320	0.467	0.000	1.000
	between		0.423	0.000	1.000		0.391	0.000	1.000
	within		0.295	-0.433	1.167		0.310	-0.480	1.120
No change_future	overall	0.490	0.500	0.000	1.000	0.522	0.500	0.000	1.000
	between		0.411	0.000	1.000		0.391	0.000	1.000
	within		0.341	-0.310	1.290		0.360	-0.278	1.322
Better off_future	overall	0.142	0.349	0.000	1.000	0.158	0.365	0.000	1.000
	between		0.266	0.000	1.000		0.279	0.000	1.000
	within		0.246	-0.658	0.942		0.263	-0.642	0.958
Age	overall	40.751	12.033	18.000	65.000	41.742	13.824	18.000	65.000
	between		12.491	18.000	65.000		14.584	18.000	65.000
	within		1.231	28.751	56.751		1.239	35.742	46.742
Age squared	overall	1805.461	1006.062	324.000	4225.000	1933.491	1154.206	324.000	4225.000
	between		1024.477	324.000	4225.000		1202.750	324.000	4225.000
	within		108.148	635.461	3435.461		110.866	1336.824	2447.824
Marital status									
With a partner who is not unemployed	overall	0.582	0.493	0.000	1.000	0.528	0.499	0.000	1.000
	between		0.494	0.000	1.000		0.499	0.000	1.000
	within		0.062	-0.168	1.249		0.041	-0.139	1.195
With an unemployed partner	overall	0.042	0.200	0.000	1.000	0.023	0.149	0.000	1.000
	between		0.197	0.000	1.000		0.149	0.000	1.000
	within		0.054	-0.625	0.792		0.034	-0.644	0.690
Single	overall	0.251	0.439	0.000	1.000	0.326	0.469	0.000	1.000
	between		0.438	0.000	1.000		0.467	0.000	1.000
	within		0.094	-0.586	1.014		0.023	-0.340	0.993
Widowed or divorced	overall	0.125	0.218	0.000	1.000	0.123	0.247	0.000	1.000
	between		0.203	0.000	1.000		0.227	0.000	1.000
	within		0.075	-0.750	0.850		0.081	-0.735	0.865
Qualification									
Degree	overall	0.386	0.487	0.000	1.000	0.246	0.431	0.000	1.000
	between		0.483	0.000	1.000		0.411	0.000	1.000
	within		0.073	-0.414	1.186		0.078	-0.554	1.046
Other degree	overall	0.091	0.287	0.000	1.000	0.107	0.309	0.000	1.000
	between		0.284	0.000	1.000		0.294	0.000	1.000

	within		0.060	-0.709	0.891		0.063	-0.693	0.907
A-level	overall	0.158	0.365	0.000	1.000	0.258	0.437	0.000	1.000
	between		0.359	0.000	1.000		0.434	0.000	1.000
	within		0.083	-0.642	0.958		0.103	-0.542	1.058
GCSE	overall	0.122	0.327	0.000	1.000	0.218	0.413	0.000	1.000
	between		0.314	0.000	1.000		0.412	0.000	1.000
	within		0.075	-0.678	0.922		0.084	-0.582	1.018
Other qualifications	overall	0.104	0.306	0.000	1.000	0.089	0.285	0.000	1.000
	between		0.295	0.000	1.000		0.278	0.000	1.000
	within		0.073	-0.696	0.904		0.064	-0.711	0.889
No qualification	overall	0.139	0.346	0.000	1.000	0.081	0.273	0.000	1.000
	between		0.347	0.000	1.000		0.281	0.000	1.000
	within		0.072	-0.661	0.939		0.054	-0.719	0.881
Unemployment benefits	overall	0.039	0.193	0.000	1.000	0.047	0.212	0.000	1.000
	between		0.161	0.000	1.000		0.190	0.000	1.000
Physical health	overall	51.285	9.462	6.560	73.120		0.138	-0.753	0.847
	between		8.785	11.108	71.220	51.531	9.703	4.480	73.040
	within		4.568	22.045	78.890		8.924	7.020	72.940
Household type							4.517	15.659	79.981
Working couple/ no child	overall	0.112	0.315	0.000	1.000	0.187	0.390	0.000	1.000
	between		0.290	0.000	1.000		0.353	0.000	1.000
	within		0.142	-0.688	0.912		0.169	-0.613	0.987
Single hh	overall	0.106	0.308	0.000	1.000	0.120	0.325	0.000	1.000
	between		0.293	0.000	1.000		0.295	0.000	1.000
	within		0.110	-0.694	0.906		0.116	-0.680	0.920
Lone Parent hh	overall	0.008	0.087	0.000	1.000	0.011	0.103	0.000	1.000
	between		0.079	0.000	1.000		0.102	0.000	1.000
	within		0.044	-0.792	0.808		0.055	-0.789	0.811
Senior couple/no child	overall	0.017	0.128	0.000	1.000	0.062	0.242	0.000	1.000
	between		0.121	0.000	1.000		0.234	0.000	1.000
	within		0.052	-0.783	0.817		0.096	-0.738	0.862
Couple w/h 1 child	overall	0.113	0.316	0.000	1.000	0.095	0.293	0.000	1.000
	between		0.271	0.000	1.000		0.255	0.000	1.000
	within		0.156	-0.687	0.913		0.151	-0.705	0.895
Couple w/h 2 children	overall	0.140	0.347	0.000	1.000	0.126	0.331	0.000	1.000
	between		0.299	0.000	1.000		0.290	0.000	1.000
	within		0.154	-0.660	0.940		0.145	-0.674	0.926
Couple w/h 3 children	overall	0.115	0.319	0.000	1.000	0.054	0.226	0.000	1.000

	between		0.289	0.000	1.000		0.204	0.000	1.000
	within		0.123	-0.685	0.915		0.096	-0.746	0.854
Others	overall	0.390	0.488	0.000	1.000	0.346	0.476	0.000	1.000
	between		0.468	0.000	1.000		0.453	0.000	1.000
	within		0.194	-0.410	1.190		0.199	-0.454	1.146
